# Aberrant STAT3 activation and overproduction of IL-21 in systemic lupus erythematosus: role of miR-155 and miR-21 in target genes *SOCS1, PTEN* and *PIAS3*

**DOI:** 10.3389/fimmu.2026.1664409

**Published:** 2026-02-02

**Authors:** Noemí Espinoza-García, Claudia Azucena Palafox-Sánchez, Adrián Ramírez De Arellano, Diana Celeste Salazar-Camarena, Katya Rocío Félix-Murray, Miguel Marín-Rosales, Pablo C. Ortiz-Lazareno, Gabriel Vega-Cornejo, Juan Armendariz-Borunda, José Francisco Muñoz-Valle

**Affiliations:** 1 Instituto de Biología Molecular en Medicina y Terapia Génica, Departamento de Biología Molecular y Genómica, Centro Universitario de Ciencias de la Salud, Universidad de Guadalajara, Guadalajara, Jalisco, Mexico; 2 Departamento de Disciplinas Filosófico, Metodológicas e Instrumentales, Centro Universitario de Ciencias de la Salud, Universidad de Guadalajara, Guadalajara, Jalisco, Mexico; 3 Instituto de Investigación en Ciencias Biomédicas (IICB), Departamento de Clínicas Médicas, Centro Universitario de Ciencias de la Salud, Universidad de Guadalajara, Guadalajara, Jalisco, Mexico; 4 Grupo de Inmunología Molecular, Centro Universitario de Ciencias de la Salud, Universidad de Guadalajara, Guadalajara, Jalisco, Mexico; 5 Doctorado en Ciencias en Biología Molecular en Medicina, Centro Universitario de Ciencias de la Salud, Universidad de Guadalajara, Guadalajara, Jalisco, Mexico; 6 Hospital General de Occidente, Secretaría de Salud Jalisco, Guadalajara, Jalisco, Mexico; 7 Centro de Investigación Biomédica de Occidente (CIBO), Instituto Mexicano del Seguro Social, Guadalajara, Jalisco, Mexico; 8 Tecnologico de Monterrey, Escuela de Medicina y Ciencias de la Salud, Guadalajara, Mexico

**Keywords:** IL-21, miR-155, miR-21, PIAS3, PTEN, SOCS1, STAT3, systemic lupus erythematosus

## Abstract

**Introduction:**

SLE is a chronic autoimmune disease characterized by immune system dysregulation, including aberrant activation of B and T lymphocytes and overproduction of proinflammatory cytokines such as IL-21. Through the STAT3 signaling pathway, this cytokine plays a key role in SLE-promoting autoantibody production and immune imbalance. It has been reported that miRNAs, such as miR-155 and miR-21, could be overexpressed in SLE and contribute to the STAT3 pathway dysregulation. We aimed to analyze the association between miR-155 and miR-21 and the expression of *SOCS1*, *PTEN*, *PIAS3*, and *IL21* in PBMC from SLE patients.

**Materials and methods:**

PBMC isolation was performed by density gradient centrifugation using Histopaque-1077, culture overnight, and seeded at a concentration of 1x10^6^ cells/mL in 24-well flat-bottom cell culture plates for subsequent stimulation with 0.5 μg/mL ionomycin and 2.5 μg/mL PMA. The expression levels of miR-155, miR-21, *SOCS1, PTEN, PIAS3*, and *IL21* were measured using the RT-qPCR technique. Western blotting determined the expression of SOCS1, PTEN, PIAS3, IL-21, and p-STAT3 proteins. IL-17A levels in cell culture supernatant were determined using ELISA to assess cell stimulation.

**Results:**

Our results showed an increased expression of miR-155 and miR-21 in SLE patients compared to HC in both, stimulated and non-stimulated PBMC. The increased miR-155 and miR-21 expression were associated with a decreased gene expression of *SOCS1* and *PTEN*. The *IL21* expression was observed in stimulated PBMC with higher levels in SLE patients. These also showed lower expression of SOCS1, PTEN, and PIAS3, while levels of IL-21 were increased in total protein from PBMC, culture cell supernatants and plasma levels. Overall, p-STAT3 was increased in the PBMC of SLE patients. Finally, miR-21 inversely correlated with *PIAS3* and *PTEN* and miR-155 with SOCS1.

**Discussion:**

These findings highlight the association between miR-155 and miR-21 with target genes SOCS1, PTEN, and PIAS3, that may contribute to the aberrant activation of the STAT3 pathway and the overproduction of IL-21 in SLE patients.

## 1 Introduction

Systemic lupus erythematosus (SLE) is a chronic, inflammatory, multisystem autoimmune disease (1). Activation of the immune system in SLE is characterized by an exaggerated B and T lymphocyte response and a loss of immune tolerance to self-antigens affecting multiple organs and systems (2). Defective antibody production and clearance, circulating and tissue immune complexes deposition, complement activation, and proinflammatory cytokines contribute to the pathogenesis of SLE (2). Thus, the expansion and survival of T and B lymphocytes, together with the maintenance of germinal centers (GC) to generate long-lived autoantibody-producing plasma cells, are essential in the pathophysiology of SLE (3, 4).

Interleukin 21 (IL-21) is a proinflammatory cytokine produced mainly by T follicular helper (Tfh), circulating T follicular helper (cTfh), T peripheral helper (Tph), T helper 17 (Th17), and TNK cells (5, 6), which are directly involved in the pathogenesis of SLE. IL-21 is a pleiotropic cytokine that affects innate and adaptive immune cells. It promotes proliferation, GC formation and maintenance, and differentiation of T cells and autoantibody-producing plasma cells (7). Its effects are mediated by the IL-21 receptor (IL-21R) through the signal transducer and activator of the transcription 3 (STAT3) signaling pathway (7).

MicroRNAs (miRNAs) are post-transcriptional regulators of gene expression, which act by base pairing to initiate the degradation or inhibit the translation of the mRNAs of their target genes (8). They are involved in many biological processes, such as cell differentiation, development, signaling, and immunity, such as microRNA-155 (miR-155) and microRNA-21 (miR-21) associated with inflammatory responses in various autoimmune diseases, including SLE (9, 10).

These miRNAs affect the expression of genes such as suppressor of cytokine signaling 1 (SOCS1), phosphatase and tensin homolog (PTEN), and protein inhibitor of activated STAT3 (PIAS3), which are negative regulators of the STAT3 signaling pathway (11), and its alteration could contribute to the immune imbalance (12–14) observed in SLE patients.

Overexpression of miR-155 and miR-21 has been observed in SLE patients (15, 16) and has been shown to target STAT3 pathway regulatory genes, SOCS1, PTEN, and PIAS3 (17–19), the main IL-21 signaling pathway (7).

In this study, we evaluated *IL21* expression in PBMC from SLE patients and the association between miR-155 and miR-21 with the expression of *SOCS1*, *PTEN*, and *PIAS3*. We analyzed how this interaction impacts the STAT3 signaling pathway in these patients. This analysis may provide further insight into the molecular regulatory mechanisms underlying SLE and potentially identify new therapeutic targets for the treatment of SLE.

## 2 Materials and methods

### 2.1 Patients and healthy controls

Fifty-eight subjects were included in this study. Thirty SLE patients and 28 HC. All patients were from the Rheumatology service of the Hospital General de Occidente, Guadalajara, México, and met the EULAR/ACR 2019 classification criteria for SLE (20). The Mexican version of the Systemic Lupus Erythematosus Disease Activity Index (Mex-SLEDAI) score (21), Systemic Lupus Erythematosus Disease Activity Index (SLEDAI-2K) (22), and the Systemic Lupus International Collaborating Clinics (SLICC) damage index (23) scores was determined in SLE patients at the time of inclusion. Clinical and immunological data were taken from medical records. This study was governed by the ethical standards and principles established in the Declaration of Helsinki and the research committees from the participant institutions (24). It was also approved by the Ethics and Research Committee from Hospital General de Occidente (no. CEI-146/21 and no. CI-146/21) and by the Universidad de Guadalajara (no. CI-02123). Before inclusion, all participants signed an informed consent form per the regulations of the General Health Law on Research for Health in Mexico.

### 2.2 PBMC isolation and stimulation

Peripheral blood mononuclear cells (PBMC) were isolated from venous blood samples collected in heparin tubes. PBMC isolation was performed by density gradient centrifugation using Histopaque-1077 (Sigma-Aldrich, Merck, Darmstadt, Germany). Washed cells were resuspended in RPMI 1640 culture medium (Gibco, Waltham, MA, USA) supplemented with 10% FCS (Gibco™, Waltham, MA, USA) and 1% penicillin/streptomycin (Gibco™, Waltham, MA, USA). PBMC were seeded at 1x10^6^ cells/mL in 24-well flat-bottom cell culture plates (Biologix, Shandong, China). PBMC were cultured for 16 hours at 37°C with 5% CO_2_. After incubation, the culture medium was removed and replaced with fresh medium before stimulation with 0.5 μg/mL ionomycin (IONO, Sigma-Aldrich, Merck, Darmstadt, Germany) and 2.5 μg/mL phorbol myristate acetate (PMA, Sigma-Aldrich, Merck, Darmstadt, Germany). Cells were divided into four experimental groups: SLE PBMC stimulated with 0.5μg/mL ionomycin and 2.5μg/mL PMA, SLE PBMC non-stimulated (cultured under the same conditions without stimulants), HC PBMC stimulated, and HC PBMC non-stimulated. Subsequently, 1x10^6^ cells/mL were incubated at 37°C with 5% CO_2_ for 3 hours for quantification of mRNA expression analysis and for 24 hours for protein quantification by Western blot. Cell-free supernatants were collected after the 24-hour incubation to assess IL-17A concentration and were stored at -40°C until the time of assay.

### 2.3 RNA extraction and quantitative reverse transcription polymerase chain reaction

To analyze mRNA and miRNA expression levels, total RNA was extracted from PBMC pellets with TRIzol reagent (Invitrogen Life Technologies, Carlsbad, CA, USA) as described in the manufacturer’s protocols. RNA was extracted from 1x10^6^ cells per condition. RNA concentration and purity were confirmed by relative absorbance at 260 nm and measurement of the 260/280 nm ratio using a NanoDrop lite spectrometer (Thermo Fisher Scientific, Waltham, MA, USA). Reverse transcription of miRNAs was performed using the TaqMan Advanced miRNA Assay cDNA synthesis kit (A28007, Thermo Fisher Scientific, Waltham, MA, USA) following the manufacturer’s instructions. We quantified the level of miR-155 and miR-21 by RT-qPCR, using TaqMan probes (Applied Biosystems, Foster City, CA, USA): miR-155 (Hs01374569_m1) and miR-21 (Hs0423142424_s1). 2 µg total RNA/sample was used. Polyadenylation reaction conditions were at 37°C for 45 minutes and 65°C for 10 minutes, followed by the adapter ligation reaction at 16°C for 60 minutes and retrotranscription at 42°C for 15 minutes, with a final incubation at 85°C for 5 minutes. The amplification reaction (miR-Amp) was carried out at 95°C for 3 seconds for 14 cycles of denaturation, 1 cycle at 60°C for 30 seconds for alignment, and 1 cycle of final reaction at 99°C for 10 minutes. RT-qPCR using TaqMan Fast Advanced Master Mix (Applied Biosystems, Foster City, CA, USA) was performed with enzymatic activation at 95°C for 20 seconds, followed by 40 cycles of denaturation at 95°C for 1 second and alignment at 60°C for 20 seconds. For quantification of *SOCS1, PTEN, PIAS3* and *IL21* genes, 2 μg of total RNA was used for reverse transcription with the M-MLV-Oligo (dT) cDNA synthesis kit (Vivantis: cat. No. RTPL12, Selangor Darul Ehsan, Malaysia), by performing a series of incubations at 65°C for 5 minutes, 42°C for 60 minutes and 85°C for 5 minutes. Samples were stored at -80°C until use. TaqMan probes (Applied Biosystems, Foster City, CA, USA) were used for qPCR: SOCS1 (Hs00705164_s1), PTEN (Hs0262621230_s1), PIAS3 (Hs00966035_m1) and *IL21* (Hs00222327_m1), using the following incubation conditions: 50°C for 2 minutes, 95°C for 20 seconds and 40 cycles of 95°C for 1 second and 60°C for 20 seconds. RT-qPCR was performed on a real-time thermal cycler (Quant Studio 5, Applied Biosystem, Foster City, CA, USA). The miR-320a was used as a standard internal reference for miR-155 and miR-21 expression, and *GAPDH* for *SOCS1, PTEN, PIAS3*, and *IL21* expression. Each sample was analyzed in duplicate. Ct values over 40 were excluded from the analysis (considered less reliable).

### 2.4 Protein isolation and western blot

We collected all cells 24 h after stimulation. Total proteins were extracted from 4x10^6^ cells using 1X RIPA lysis buffer (Sigma-Aldrich, Merck, Darmstadt, Germany) supplemented with a combination of protease and phosphatase inhibitors (ABCAM, Cambridge, UK). Coomassie blue staining with Bradford’s reagent (Sigma-Aldrich, Merck, Darmstadt, Germany) was performed to quantify the proteins. Twenty µg of total protein was separated on a 10% sodium dodecyl sulfate polyacrylamide gel electrophoresis (SDS-PAGE) gel under reducing conditions and then transferred to a polyvinylidene difluoride (PVDF) membrane (Bio-Rad Laboratories, CA, USA), which was blocked with 5% milk powder containing 0.1% Tween 20 at 4°C for 2 hours. Subsequently, the membranes were immunoblotted with the specific primary antibodies, anti-IL-21 (ABCAM, Cambridge, UK) (1:3000), phosphorylated STAT3 (p-STAT3) on tyrosine 705 (Y705) (Santa Cruz Biotechnology, Dallas, TX, USA) (1:400), SOCS1 (Santa Cruz Biotechnology, Dallas, TX, USA) (1:500), PTEN (Santa Cruz Biotechnology, Dallas, TX, USA) (1:500) and PIAS3 (Santa Cruz Biotechnology, Dallas, TX, USA) (1:200) overnight at 4°C. The membranes were then incubated with horseradish peroxidase (HRP)-conjugated secondary antibody, anti-mouse m-IgG (Santa Cruz Biotechnology, Dallas, TX, USA) (1:2500), and anti-rabbit IgG (ABCAM, Cambridge, UK) (1:5000) for 1.5 hours at room temperature under agitation. The membranes were then developed using Immobilon HRP substrate for immunoelectrotransfer (Sigma-Aldrich, Merck, Darmstadt, Germany), and images were captured using a Digital Chemiluminescence System (MicroChemi 4.2, Jackson, USA). The grey intensity value of each protein band was analyzed using GelQuant.NET software (BiochemLabSolutions) and each value was normalized to the corresponding control protein band. β-actin (Santa Cruz Biotechnology, Dallas, TX, USA) (1:5000) was used as a loading control.

### 2.5 ELISA

Plasma was separated from the total blood of each subject included in this study at the time of sample collection and stored at -20°C until use. IL-21 levels concentrations were determined using the ELISA MAX™ Deluxe Set Human IL-21 (cat. No 433804, BioLegend, CA, USA), while IL-17A concentration was quantified using the Human IL-17 Quantikine ELISA Kit (cat No. D1700, R&D systems, Minneapolis, MN, USA), following the manufacturer’s instructions. All assays were performed in duplicate. Optical density was measured at 450 and 570 nm using a Multiskan™ GO microplate reader (Thermo Fisher Scientific). Cytokine concentrations were calculated from a standard curve and expressed in pg/mL.

### 2.6 Statistical analysis

Statistical analyses were performed using software packages from IBM SPSS statistics v25 (IBM Corporation; Armonk, NY, USA) and GraphPad Prism v10.2.3 (GraphPad Software Incorporation; La Jolla, CA, USA). Shapiro–Wilk test was used to determine normality distribution. Categorical variables are absolute values and percentages, whereas continuous variables are medians and 25th-75th percentiles. Mann–Whitney U test was used to compare differences between the two groups, and Kruskal–Wallis with Dunn’s test as *post hoc* for multiple comparisons. Spearman’s test was used to calculate correlations. p ≤ 0.05 values were considered statistically significant. ANCOVA was used for multiple linear regression. We used the instrument’s default threshold settings for each RT-qPCR run to calculate CT. We used the comparative CT method (2^-ΔΔCt^) to calculate changes in mRNA expression and 2^-ΔCt^ to calculate relative mRNA and miRNA expression.

## 3 Results

### 3.1 Demographic and clinical characteristics

A total of 58 participants, including 30 SLE patients and 28 HC, were included in this study. All females with a median age of 40 (IQR 27-52) for SLE and 26 (IQR 24-30) years old for HC. Disease evolution was 10 (IQR 0.25-31) years. The median disease activity scores were 1 (0–30) for SLEDAI-2K and 1.5 (0-14) for Mex-SLEDAI, 90% of SLE patients were inactive/mildly active (SLEDAI-2K score 0-5). Additionally, most patients had no damage, with a median score of 0 (IQR 0-2) according to the SLICC damage index. The frequency of clinical manifestations and ongoing treatment are shown in **Table 1**.

**Table 1:** Clinical and demographic characteristics in SLE patients.

Variable	SLE (*n* = 30)
Age, years (IQR)	40 (27-52)
Female gender, *n* (%)	30 (100)
Disease duration, years (min-max)	10 (0.25-31)
Family history of rheumatic disease, *n* (%)	13/30 (43.3)
Mex-SLEDAI score (min-max)	1.5 (0-14)
Inactive, *n* (%)	15/30 (50.0)
Active, *n* (%)	15/30 (50.0)
SLEDAI-2K score (min-max)	1 (0-30)
Inactive/mildly active	27/30 (90.0)
Clearly active	3/30 (10.0)
SLICC score (min-max)	0 (0-2)
Non-Damage *n* (%)	26/30 (86.7)
Damage, *n* (%)	4/30 (13.3)
Autoantibody profile	
Antinuclear antibodies (ANAs), *n* (%)	30/30 (100)
ANAs Titles	1:1280 (1:320-1:1280)
Anti-dsDNA+, *n* (%)	8/20 (40.0)
Anti-RNP+, *n* (%)	5/30 (16.7)
Anti-Ro+, *n* (%)	2/30 (6.7)
Anti-Sm+, *n* (%)	2/30 (6.7)
Clinical domains	
Mucocutaneous^a^, *n* (%)	10/30 (33.3)
Hematological^b^, *n* (%)	5/30 (16.7)
Renal^c^, *n* (%)	4/30 (13.3)
Musculoskeletal^d^, *n* (%)	3/30 (10.0)
Treatment	
Untreatment, *n* (%)	2/30 (6.7)
Antimalarial, *n* (%)	18/30 (60.0)
Prednisone, *n* (%)	15/30 (50.0)
Prednisone dose	5.9 (0.0-75.0)
Azathioprine, *n* (%)	10/30 (33.3)
Mycophenolate Mofetil, *n* (%)	8/30 (26.7)
Metotrexate, *n* (%)	4/30 (13.3)
Cyclophosphamide, *n* (%)	1 (3.3)

Mex-SLEDAI: inactive (score 0-1), Active (score ≥2); SLEDAI-2K: Inactive/mildly active (score <6), Clearly active (score ≥6); SLICC: Non-damage (SLICC score 0), damage (SLICC score >1); ^a^Mucocutaneous: malar erythema, alopecia, oral ulcers, and photosensitivity, ^b^Hematologic: leukopenia, lymphopenia and thrombocytopenia, ^c^Renal: persistent proteinuria (>0.5 g/día) and cylinders, ^d^Musculoskeletal: arthritis. ANAs, antinuclear antibodies; SLE, systemic lupus erythematosus; max, maximum; min, minimum. Mex-SLEDAI, Mexican version of the Systemic Lupus Erythematosus Disease Activity Index; SLEDAI-2K, Systemic Lupus Erythematosus Disease Activity Index 2000; SLICC, Systemic Lupus International Collaborating Clinics.

### 3.2 Increased expression of miR-155 and miR-21 is associated with reduced levels of SOCS1, PTEN, and PIAS3 in the IL-21 signaling pathway

**Figure 1:**
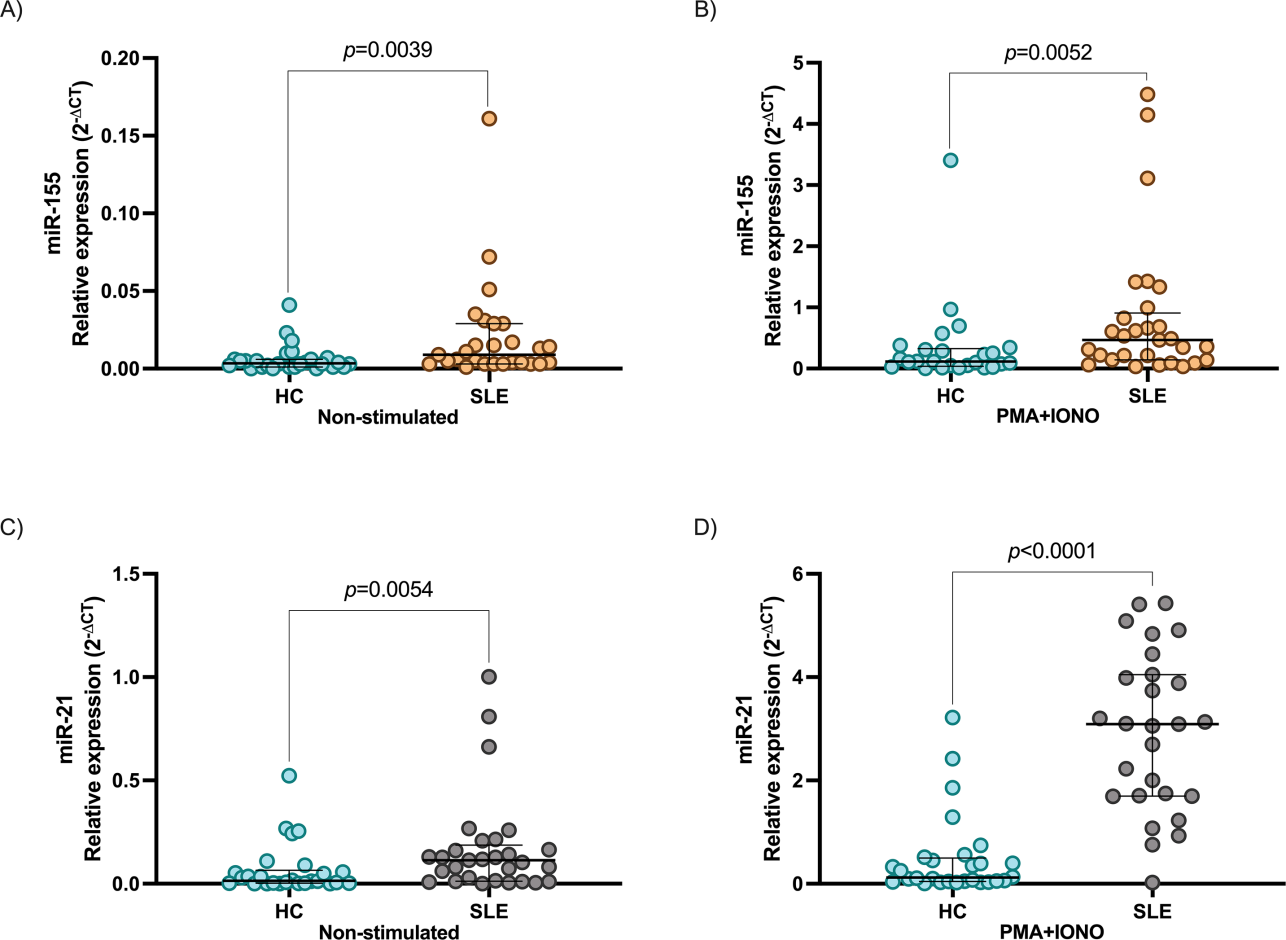
Expression of miR-155 and miR-21 in PBMC between SLE patients and healthy controls. **(A)** miR-155 expression between HC and SLE patients non-stimulated, **(B)** miR-155 expression between HC and SLE patients after stimulation with PMA+IONO, **(C)** miR-21 expression between HC and SLE patients non-stimulated, **(D)** miR-21 expression between HC and SLE patients after stimulation with PMA+IONO. Data are shown in median and IQR. p-value was obtained using Mann–Whitney’s U test. The 2^−ΔCt^ method was used to analyze changes in miRNA expression levels. miR-320a served as internal reference normalized for miR-155, miR-21.

**Figure 2:**
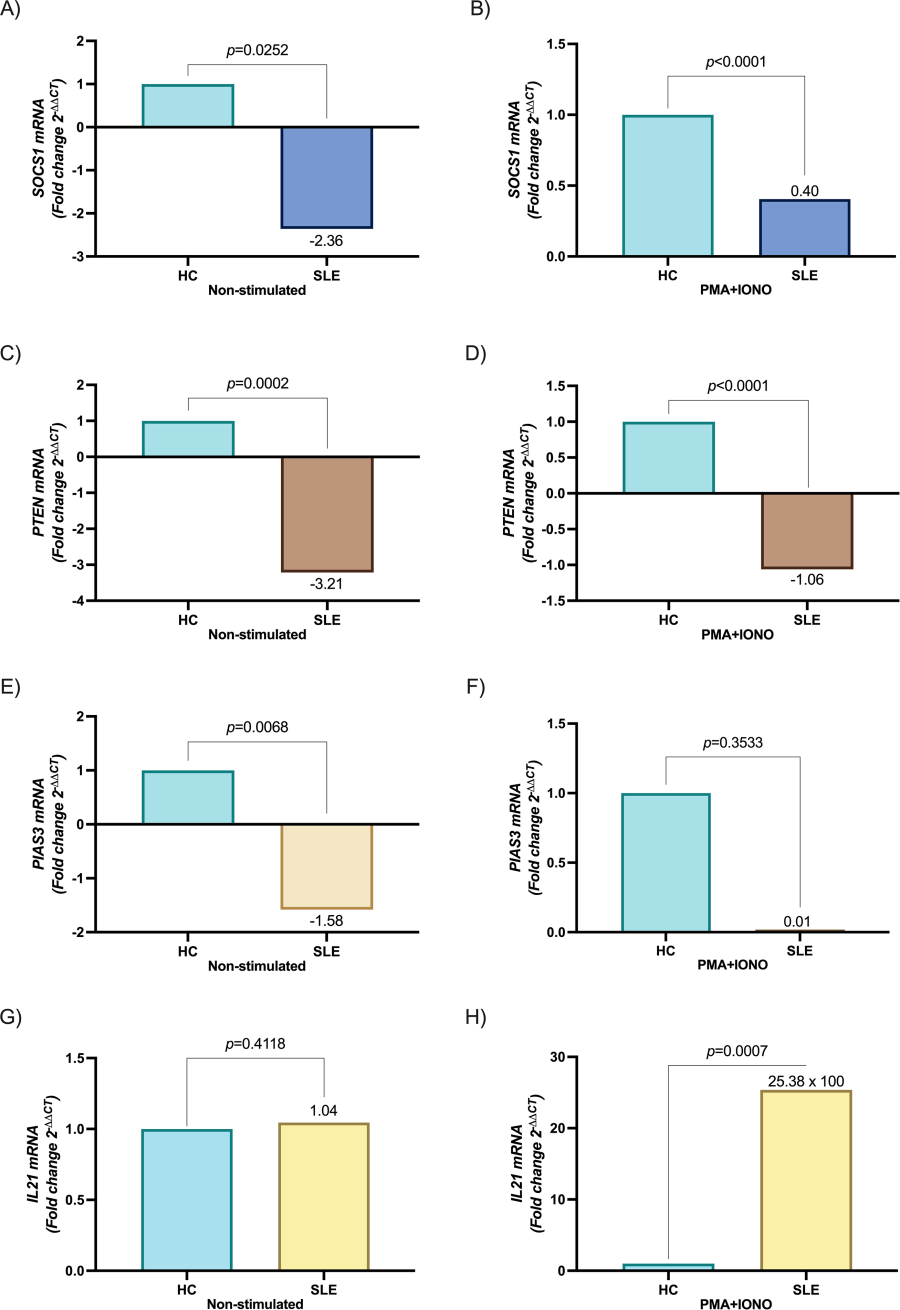
Expression of genes *SOCS1, PTEN*, and *PIAS3* in PBMC between SLE patients and healthy controls. **(A)***SOCS1* mRNA expression between HC and SLE patients non-stimulated, **(B)***SOCS1* mRNA expression between HC and SLE patients after stimulation with PMA+IONO, **(C)***PTEN* mRNA expression between HC and SLE patients non-stimulated, **(D)***PTEN* mRNA expression between HC and SLE patients after stimulation with PMA+IONO, **(E)***PIAS3* mRNA expression between HC and SLE patients non-stimulated, **(F)***PIAS3* mRNA expression between HC and SLE patients after stimulation with PMA+IONO, **(G)***IL21* mRNA expression between HC and SLE patients non-stimulated, **(H)***IL21* mRNA expression between HC and SLE patients after stimulation with PMA+IONO. Data are shown in median and IQR. p-value was obtained using Mann–Whitney’s U test. Fold change values (2^-ΔΔCT^) are relative to healthy controls in mRNA expression. *GAPDH* served as internal reference normalized for *SOCS1* mRNA, *PTEN* mRNA, *PIAS3* mRNA and *IL21* mRNA.

To analyze the impact of miR-155 and miR-21 on the regulatory molecules of IL-21 signaling pathways, we evaluated the expression of miR-21, miR-155, and *SOCS1, PTEN, PIAS3*, and *IL21* in stimulated and non-stimulated PBMC from SLE patients and HC. The miR-155 and miR-21 levels were significantly higher in PBMC from SLE patients than HC (*p* = 0.0039 and *p* = 0.0054, respectively, **Figures 1A, B**). miR-155 and miR-21 expression was increased after PMA+IONO stimulation in both groups, being higher in SLE patients (*p* = 0.0052 and *p* < 0.0001, respectively, **Figures 1C, D**). In concordance with this *SOCS1* and *PTEN* were downregulated in stimulated and non-stimulated PBMC from SLE patients (*p* < 0.0001 and *p* < 0.0001; *p* = 0.0252 and *p* = 0.0002, respectively, **Figures 2A-D**). The difference in *PIAS3* expression was not maintained after the stimulus (p=0.0068 and p=0.3533, respectively, **Figures 2E, F**). When we evaluated non-stimulated PBMC, *IL21* expression was not observed in SLE patients and HC (*p* = 0.4118, **Figure 2G**). In contrast, after stimulation with PMA+IONO a significant increase in *IL21* expression was observed in SLE patients compared to HC (*p* = 0.0007, **Figure 2H**).

### 3.3 Increased miR-155 and miR-21 levels associates with reduced expression of target genes *SOCS1, PTEN*, and *PIAS3*

Using computational prediction tools, we identified that *SOCS1* and *PTEN* are targets of miR-155 and *SOCS1, PTEN* and *PIAS3* of miR-21. Target prediction was performed using miRTargetLink 2.0, miRCarta, miRPathDB v2.0, miRBase, miRWalk, TargetScanHuman, miRDB, miRTarBase, and RNAHybrid, based on sequence complementarity and conservation across species. To determine the biological significance of SOCS1/PTEN-miR-155 and SOCS1/PTEN/PIAS3-miR-21 interaction in SLE, we compared the expression between the miRNAs and their target genes in PBMC from SLE patients and HC. We hypothesized that overexpression of miR-155 and miR-21 in SLE might result from reduced SOCS1, PTEN, and PIAS3 expression.

### 3.4 SOCS1, PTEN, and PIAS3 expression is decreased in PBMC from SLE patients and provides possible positive feedback of IL-21 through p-STAT3.

**Figure 3:**
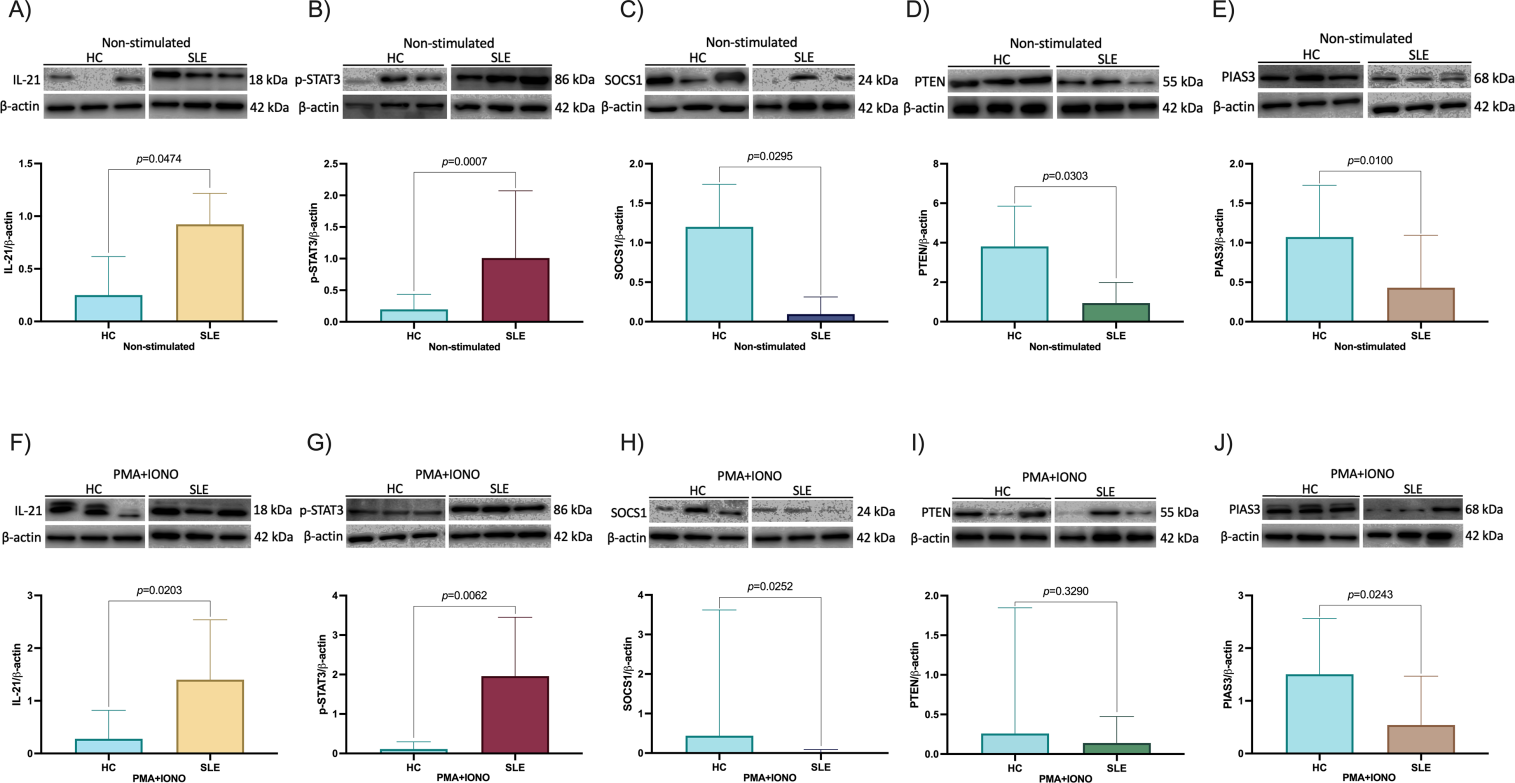
Protein expression of IL-21, p-STAT3, SOCS1, PTEN and PIAS3. **(A)** IL-21 expression in HC and SLE patients non-stimulated, **(B)** p-STAT3 expression in HC and SLE patients non-stimulated, **(C)** SOCS1 expression in HC and SLE patients non-stimulated, **(D)** PTEN expression in HC and SLE patients non-stimulated, **(E)** PIAS3 expression in HC and SLE patients non-stimulated, **(F)** IL-21 expression after stimulation with PMA+IONO in HC and SLE patients, **(G)** p-STAT3 expression after stimulation with PMA+IONO in HC and SLE patients, **(H)** SOCS1 expression after stimulation with PMA+IONO in HC and SLE patients, **(I)** PTEN expression after stimulation with PMA+IONO in HC and SLE patients, **(J)** PIAS3 expression after stimulation with PMA+IONO in HC and SLE patients. Data are shown in median and IQR. p-value was obtained through Mann-Whitney’s U test. β-actin served as an internal reference.

To evaluate the IL-21/STAT3 signaling pathway activation in our SLE patients, we measured protein expression of IL-21, p-STAT3, SOCS1, PTEN, and PIAS3 in PBMC both stimulated and non-stimulated with PMA+IONO. Western blot results showed that PBMC from non-stimulated SLE patients had higher IL-21 protein expression compared to non-stimulated HC (*p* = 0.0474, **Figure 3A**), higher protein expression of p-STAT3 (*p* = 0.0007, **Figure 3B**), and lower protein expression of SOCS1, PTEN, and PIAS3 (*p* = 0.0295, *p* = 0.0303, *p* = 0.0100, respectively, **Figures 3C–E**). Also, after PBMC stimulation with PMA+IONO, IL-21(*p* = 0.0203, **Figure 3F**) and p-STAT3 (*p* = 0.0062, **Figure 3G**) remains higher, while SOCS1 (*p* = 0.0252, **Figure 3H**) and PIAS3 (*p* = 0.0243, **Figure 3J**) protein expression were lower compared to stimulated HC. PTEN protein expression showed no significant difference (*p* = 0.3290, **Figure 3I**).

### 3.5 Increased proinflammatory cytokines IL-21 and IL-17A in SLE patients compared to HC

**Figure 4:**
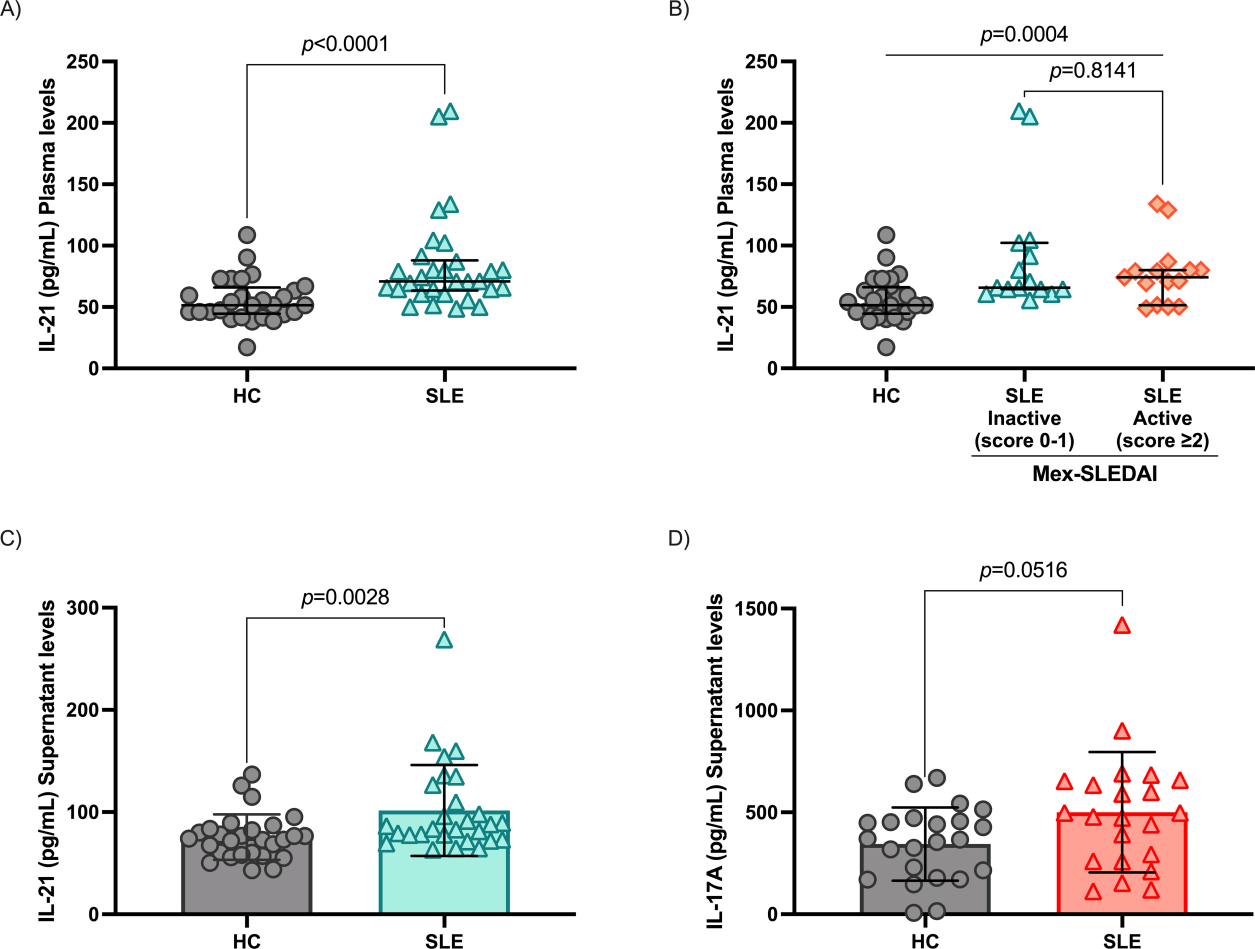
Cytokines IL-21 and IL-17A levels. **(A)** IL-21 plasma levels in HC and SLE patients, **(B)** comparison of IL-21 levels between disease activity groups according to Mex-SLEDAI, **(C)** IL-21 cytokine measurement in the cell culture supernatant of PMA+IONO-stimulated PBMC, **(D)** IL-17A levels measurement in the cell culture supernatant of PMA+IONO-stimulated PBMC. Data are shown in median and IQR. p-value was obtained through Mann-Whitney’s U test and Kruskal-Wallis with Dunn’s *post-hoc*, according to the case.

Plasma IL-21 levels were significantly elevated in SLE patients compared to HC (70.88 vs 51.47 pg/mL, respectively, *p* < 0.0001, **Figure 4A**). Interestingly, no significant differences were observed between patients with inactive and active disease (*p* = 0.8141, **Figure 4B**). Also, supernatant IL-21 and IL-17 levels were higher in SLE patients compared with HC (*p* = 0.0028, **Figure 4C** and p=0.0516, **Figure 4D**).

### 3.6 Inverse correlation between miR-155 and miR-21 and their regulatory targets in SLE

**Figure 5:**
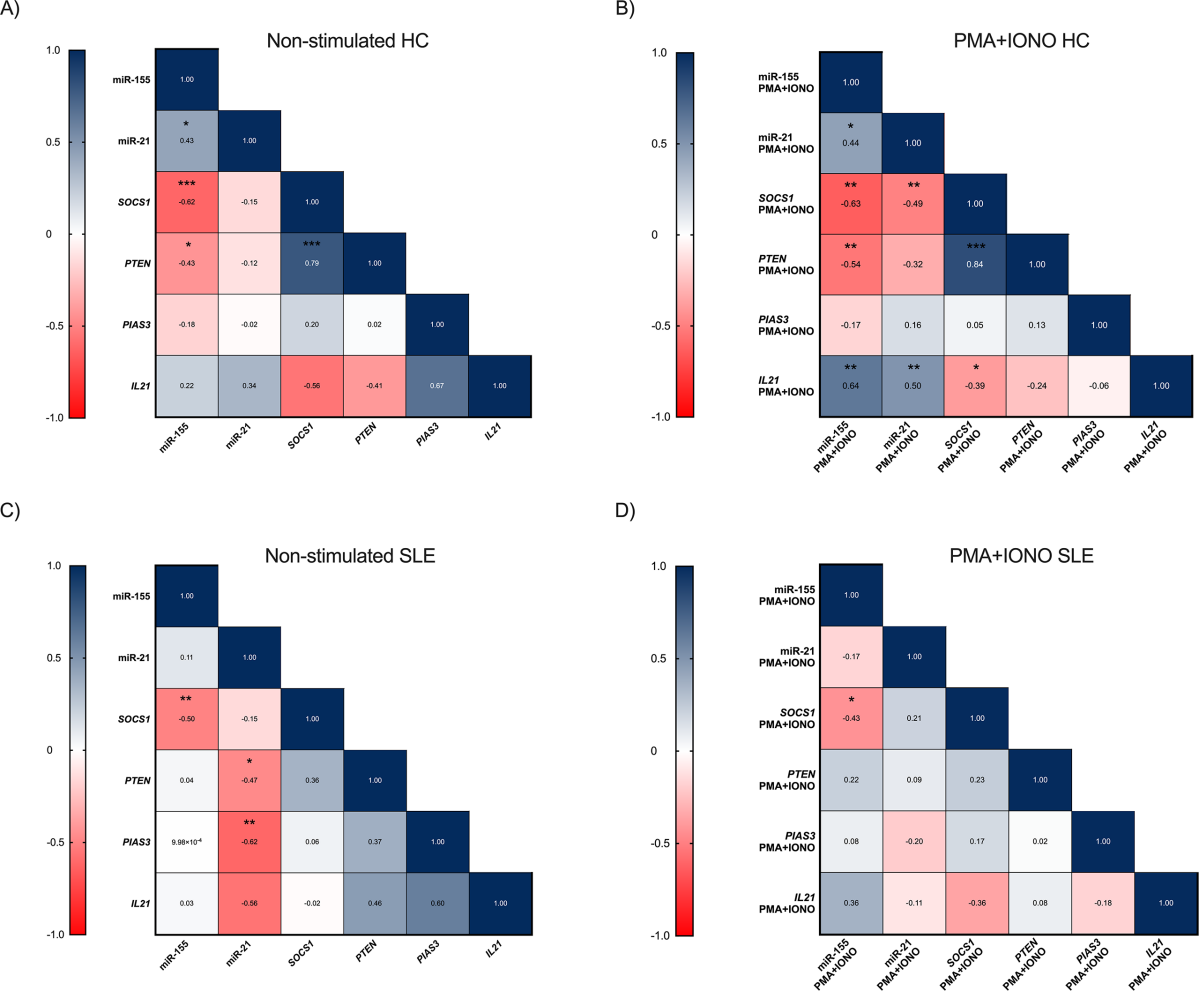
Correlation among miR-155, miR-21, *SOCS1* mRNA, *PTEN* mRNA, *PIAS3* mRNA and *IL21* mRNA in HC and SLE patients. **(A)** correlation between the miR-155, miR-21, *SOCS1* mRNA, *PTEN* mRNA, *PIAS3* mRNA and *IL21* mRNA non-stimulated in HC, **(B)** correlation between the miR-155, miR-21, *SOCS1* mRNA, *PTEN* mRNA, *PIAS3* mRNA and *IL21* mRNA after stimulation with PMA+IONO in HC, **(C)** correlation between the miR-155, miR-21, *SOCS1* mRNA, *PTEN* mRNA, *PIAS3* mRNA and *IL21* mRNA non-stimulated in SLE patients, **(D)** correlation between the miR-155, miR-21, *SOCS1* mRNA, *PTEN* mRNA, *PIAS3* mRNA and *IL21* mRNA after stimulation with PMA+IONO in SLE patients. p-value was obtained using Spearman’s correlation. *p ≤ 0.05, **p ≤ 0.001, ***p ≤ 0.0001.

In non-stimulated PBMC from HC, we found that miR-155 expression showed negative correlation with their target genes *SOCS1* (*r* = -0.62, *p* = 0.0005) and *PTEN* (*r* = -0.43, *p* = 0.0213), **Figure 5A**. After stimulation with PMA+IONO, miR-155 remained negatively correlated with *SOCS1* (*r* = -0.63, *p* = 0.0007) and *PTEN* (*r* = -0.54, *p* = 0.0058). In addition, miR-155 was positively correlated with *IL21* expression (*r* = 0.64, *p* = 0.0011). We also observed a negative correlation between miR-21 and their target gene *SOCS1* (*r* = -0.49, *p* = 0.0077) and a positive correlation with *IL21* (*r* = 0.50, *p* = 0.0088), **Figure 5B**. In non-stimulated SLE patients, a negative correlation was observed between miR-155 and their target gene *SOCS1* (*r* = -0.50, *p* = 0.0090), as well as between miR-21 and their target genes *PTEN* (*r* = -0.47, *p* = 0.0110) and *PIAS3* (*r* = -0.62, *p* = 0.0010), **Figure 5C**.

After stimulation, in SLE patients, miR-155 expression was negatively correlated with their target gene *SOCS1* (*r* = -0.43, *p* = 0.0206), **Figure 5D**.

### 3.7 Evaluation of age and pharmacological treatment as covariates through ANCOVA

To identify whether the differences observed between SLE patients and HC remained independent of age or pharmacological treatment, we performed analysis of covariance (ANCOVA) using group as the factor and age, prednisone, antimalarial and azathioprine as the covariate. In all markers evaluated, including miRNAs (miR-155, miR-21), their regulatory molecules (SOCS1, PTEN, PIAS3) and p-STAT3 before and after stimulation with PMA+IONO, and IL-21. Neither the group effect nor the age effect or antimalarial effect showed significant associations (*p*>0.05) with the levels of these variables after adjustment, indicating that, in our cohort, age does not act as a confounding factor and that the descriptive differences between groups are not explained by age or antimalarial treatment (data not shown). Prednisone showed significant associations with IL-21 protein expression (non-stimulated: VIF 1.8, β=1.557, *p* = 0.0004; PMA+IONO stimulation: VIF 1.4, β=1.660, *p* = 0.0116) and PIAS3 mRNA PMA+IONO stimulation (VIF 1.7, β=2.4, *p* = 0.0298). Azathioprine was significantly associated only with miR-21 expression non-stimulated (VIF 1.3, β=-1.868, *p* = 0.0307) and PTEN protein expression non-stimulation (VIF 2.7, β=-2.183, *p* < 0.0001). However, all VIF values remained low, indicating minimal collinearity and confirming that these treatment effects do not compromise model interpretation. After adjusting for pharmacological treatment, the group differences remained, supporting that the alterations observed in these molecules are driven by the pathology rather than by treatment effects.

## 4 Discussion

SLE is an autoimmune disease characterized by antibody production, immune complexes deposition, and proinflammatory cytokines followed by tissue damage. The activation of the immune system in SLE includes aberrant B and T lymphocyte response and a loss of immune tolerance to self-antigens, which affects multiple organs and systems (2). IL-21 is an important cytokine produced by T cell subpopulations, Tfh, cTfh, Tph, and Th17 cells (5, 6). IL-21 plays a key role in this process by activating B cells and promoting the differentiation of these cells into autoantibody-secreting plasma cells, which are characteristic features of SLE (7). IL-21 signals through the STAT3 pathway (7) are regulated at various levels by inhibitors such as SOCS1, PTEN, and PIAS3, which form a negative feedback loop attenuating signaling (17–19). Also, miRNAs play a critical role in post-transcriptional gene regulation (8), specifically miR-155 and miR-21 have been implicated in autoimmune diseases such as SLE (9, 10) by targeting the regulatory molecules of the IL-21 signaling pathway, SOCS1, PTEN, and PIAS3 (17–19).

Our study aimed to compare *IL21* expression with the expression of STAT3 signaling pathway regulatory genes in PBMC from SLE patients. Our results showed that the miR-155 and miR-21 expression were significantly increased in the PBMC from SLE patients. Similarly, previous reports observed an increased miR-155 and miR-21 expression in PBMC, peripheral blood, CD4+ T cells, exosomes, serum, and plasma of SLE patients (9, 15, 17, 25–27). However, most of these studies have not explored their functional impact on target genes and their relationship to disease.

The overexpression of miR-155 and miR-21 has been associated with immune system cell activation, proliferation (28–30), and the production of proinflammatory cytokines (31–33). miR-155 has been extensively studied and identified as a key miRNA in SLE due to its role in T cell proliferation, differentiation, and development (34, 35). Recent studies show that its overexpression contributes to B and T cell hyperactivity, promoting increased production of autoantibodies and exacerbating the inflammation characteristic of SLE (34, 35). In addition, miR-155 is critical in fate engagement with plasma cells by negatively regulating paired box gene 5 (PAX5) (36). Also, it has been reported that miR-155 controls the extent of the extrafollicular response by regulating the survival and proliferation of B blasts, plasmablasts, and antibody production (37). Chauhan et al. reported that miR-155 was positively regulated in anti-double-stranded DNA (anti-dsDNA)-positive SLE patients (38). Finally, increased miR-155 expression correlated with an increase in the frequency of peripheral double negative (DN) B cells (39), which were also reported expanded in SLE patients (40). miR-21, on the other hand, in addition to T cells, was found to be overexpressed in B cells (17). miR-21 has been shown to promote the survival and proliferation of autoreactive B cells and inhibit T cells’ regulatory function (41). Noting that there is an association between miR-21 and anti-dsDNA in SLE patients (16). Salvi et al., 2018 demonstrated that miR-21 activates type I interferon (IFN) secretion by plasmacytoid dendritic cells (pDCs) in human blood *in vitro* and that this effect is enhanced in SLE patients. It also induces the secretion of the proinflammatory cytokines TNF-α and IL-6 (42). Another study observed that miR-21 is involved in losing B cell tolerance and autoreactive B cell development, GC responses, and Ab-forming cells (AFCs) development in SLE (43).

In addition to the regulatory effects of miR-155 and miR-21, it is important to consider their interaction with key genes such as *SOCS1*, *PTEN*, and *PIAS3*, which act as critical modulators of immune responses. Our results show that SLE patients have lower mRNA and protein expression of SOCS1, PTEN, and PIAS3 than HC. Like our results, others studies reported decreased expression of SOCS1 in SLE patients (44, 45). SOCS1 expression has also been reduced in other diseases (46, 47), demonstrating that SOCS1 deficiency in T cells, activate proliferation and IFN-γ secretion, leading to disease exacerbation mediated by inhibition of the JAK2/STAT3 pathway (46). SOCS1 is a negative regulator of the STAT3 signaling pathway, playing an important role as a suppressor of inflammation (46–48). Previous studies have shown that patients with active SLE have lower SOCS1 expression than those with inactive disease (45). SOCS1 deficiency potentiates inflammation and promotes the secretion of proinflammatory cytokines, exacerbating the immune imbalance in these patients (46, 47). Takahashi et al., 2011 found that Foxp3^+^ regulatory T (Treg) cells tend to show increased plasticity and lean towards the type 1 helper T cell (Th1)/Th17 phenotype when deficient in SOCS1 (49). They observed that IFN-γ^-/-^ SOCS1^-/-^ Treg cells had hyperactivation of STAT3 and increased IL-17A production compared to IFN-γ^−/−^ SOCS1^+/+^ Treg cells. Thus, they propose that SOCS1 maintains Treg cell integrity and function by maintaining Foxp3 expression and suppressing STAT3-driven IL-17A production (49).

Decreased or loss of PTEN expression can also lead to inflammatory disorders (13), such as SLE, as it is an important regulator of the same STAT3 signaling pathway (50), promoting the survival of autoreactive cells and exacerbating the pathology of this disease (51). Like our results, Cui et al., 2017 reported that PTEN expression is significantly lower in SLE patients than in HC and was associated with lower expression in patients with active than inactive SLE (52). In addition, one study found that *PTEN* mRNA expression was decreased in the lupus nephritis group compared to that of the non-lupus nephritis group (53). Wu et al., 2014 observed that all SLE B-cell subsets, except memory B cells, showed reduced PTEN expression compared to B cells from HC. Furthermore, PTEN expression level was inversely correlated with disease activity (17). Other studies have found that PTEN overexpression reduces T-cell activation, STAT3 activity and Th17 cell differentiation by modulating the balance between Th17 and Treg (51) and may enhance Treg cell stability by suppressing Th1 and Tfh cell responses. PTEN depletion in Treg cells resulted in excessive Tfh and germinal center cell responses and spontaneous inflammatory disease (13). PTEN has also been shown to regulate Th17 cell differentiation by suppressing IL-2 production. PTEN deficiency increases IL-2 expression and STAT5 phosphorylation but reduces STAT3 phosphorylation, inhibiting Th17 cell differentiation (54). PIAS3 acts by inhibiting STAT3 pathway signaling (11); to our knowledge, this is the first report of PIAS3 in SLE patients. After stimulation, *PIAS3* mRNA expression showed no significant differences between SLE patients and HC. However, a downward trend of this regulator is observed. Like our results, there are studies in other diseases, such as glioblastoma multiforme, lung cancer, breast cancer, arthritis rheumatoid (RA), endometriosis, and psoriatic lesions, where low PIAS3 expression was observed (55, 56). At the same time, STAT3 activation increased (55). Tang et al., 2016 observed a negative correlation between *PIAS3* mRNA expression and STAT3 mRNA expression and the percentage of Th17 cells (56). Thus, reduced SOCS1, PTEN, and PIAS3 expression could result in constitutive activation of the STAT3 signaling pathway, contributing to an uncontrolled inflammatory response in SLE patients.

On the other hand, *IL21* mRNA expression was not detected in any of the study groups under basal conditions. However, the presence of IL-21 protein was observed under these conditions. After stimulation with PMA+IONO, there was a significant increase in both *IL21* mRNA and IL-21 protein expression in SLE patients. These results reflect an interesting phenomenon in the regulation of IL-21. Under basal conditions, it has been observed that SLE patients have no *IL21* mRNA expression in non-stimulated PBMC, which could be attributed to the short half-life of cytokine mRNAs, especially in the absence of specific stimuli to stabilize their transcription (57). However, the presence of IL-21 protein under these same conditions may reflect the more stable nature of the protein compared to its mRNA. This may be because once synthesized, the protein remains functional for longer periods or is protected by mechanisms that regulate its degradation depending on its function and regulation (58, 59), allowing its detection even when mRNA production is not in progress. Our results showed that, after stimulation with PMA+IONO, there was a considerable 2538-fold increase in *IL21* expression in PBMC from SLE patients compared to HC. This suggests that more mRNA is being transcribed under an inflammatory environment, as in SLE. IL-21 positively regulates its expression in peripheral blood CD3+ lymphocytes (60). This autoregulatory mechanism, common in autocrine cytokines such as IL-21, is particularly relevant in SLE, where excessive IL-21 production generates a positive feedback loop. This not only amplifies cell activation but also enhances the signaling of key pathways, such as STAT3, contributing to the pathogenesis of the disease. Also, cytokine stimulation induces Y705 STAT3 phosphorylation, while S727 is more influenced by the basal activation state of the cell (19). In addition, *IL21* mRNA has a short half-life, preventing its accumulation without stabilizing stimuli. Upon stimulation, rapid phosphorylation of Y705 triggers an increase in *IL21* transcription. This initial event is subsequently sustained by slower phosphorylation of S727, supporting prolonged IL-21 production. This regulatory mechanism may explain the ability of immune cells in SLE to respond rapidly to initial stimuli with increased IL-21 and, at the same time, maintain elevated levels for prolonged periods. Like our results, Dolff et al., 2011 observed that non-stimulated T cells from SLE patients and HC did not express IL-21 (61). On the other hand, Rasmussen et al., 2015 reported that purified, non-stimulated CD4+ T cells from SLE patients had significantly elevated levels of *IL21* mRNA compared to HC. However, after induction with IL-21, *IL21* mRNA levels were significantly increased in HC, but not in SLE patients (19). Consistent with our results, other studies have detected increased *IL21* mRNA expression in SLE patients relative to HC upon adding a stimulus (62, 63). Furthermore, Nakou et al., 2013 found that patients with active SLE had 4-fold higher *IL21* mRNA than patients with inactive SLE and HC (64). Studies in other diseases also reported higher *IL21* mRNA expression in patients than HC (65–67).

Previous studies have shown that IL-21 overexpression contributes to the generation of memory and plasma B cells (64), and high concentrations of IL-21 are associated with disease severity, such as SLEDAI scores (64, 68). In addition, IL-21 can potently induce CD11c^hi^ T-bet+ B cells and promote the differentiation of these cells into autoantibody-secreting autoreactive plasma cells (69). IL-21 can promote autoantibody production by inducing IgG class switch recombination in B cells (70). Another study showed that IL-21 is important for the maturation of T and B cells in the GC and supports the formation of antigen-specific memory B cells and plasma cells (71). Studies in murine models of MRL-Faslpr lupus have shown that blockade of IL-21 with IL-21R/Fc can attenuate B-cell hyperactivity as well as the aberrant Tfh cell pathway that contributes to SLE pathogenesis (72, 73). These observations are consistent with a role for IL-21 in plasma cell differentiation and susceptibility to develop SLE, contributing to the persistence of the autoimmune response and immune complex-mediated tissue damage. This cytokine also plays a crucial role in the lineage commitment of Th17 cells, known for their proinflammatory role (65) and high production of IL-21 and IL-17 (74, 75). IL-21, in combination with other cytokines such as IL-6 and TGF-β, promotes Th17 cell differentiation from naïve precursors via the STAT3 pathway (76, 77).

STAT3 expression in SLE reveals key patterns of activation and regulation that contribute to the pathophysiology of the disease. Our results show that p-STAT3 expression levels are significantly higher in SLE patients, both under basal conditions and after stimulation with PMA+IONO. This suggests a sustained activation and increased sensitivity of the STAT3 signaling pathway in SLE patients, reflecting chronic immune hyperactivity characteristic of autoimmune diseases. STAT3 activation is related to the overexpression of cytokines such as IL-6, IL-10, and IL-21, which are abundant in the SLE microenvironment and act directly on this pathway (40, 78–80). Under basal conditions, this constitutive activation could maintain a persistent inflammatory state, contributing to the generation of aberrant immune responses. Upon cell stimulation, further upregulation of p-STAT3 evidence increased sensitivity to external stimuli, exacerbating inflammation and favoring the production of autoantibodies, findings that have been previously reported (48, 81). Constitutive STAT3 activation plays a crucial role in developing proinflammatory cell subpopulations. For example, the differentiation of Tfh and Th17 cells, which are critical in the pathogenesis of SLE, depends on this pathway (82). In addition, STAT3 negatively regulates Treg cells, which act to restrain pathological immune responses (83). It has been proposed that IL-23/IL-23R expression is favorably regulated in SLE (82) and that enhanced IL-23 expression is mainly responsible for the positive regulation of STAT3 (48).

Consistent with our results, other studies have found increased STAT3 and p-STAT3 expression levels in SLE patients (84, 85), observing a significantly higher translocation of p-STAT3 to the nucleus of SLE T cells (84). This phenomenon could be linked to the persistence of chronic inflammation and the positive regulation of target genes such as *IL21*, which amplifies the signaling pathway through an autocrine loop (60, 66). Furthermore, STAT3 deletion in CD4+ T cells has been shown to drastically reduce IL-21 and IL-17 expression, B-cell activation, autoantibody production, and cellular infiltration of the kidneys, highlighting its central role in SLE pathogenesis (48, 86). Under normal conditions, some mechanisms negatively regulate STAT3 activation, such as SOCS1, PTEN, and PIAS3 proteins, which limit its activity and prevent excessive signaling (51, 87, 88). However, we demonstrated that these mechanisms may be insufficient in SLE, allowing uncontrolled STAT3 activation. Our results suggest that SLE is driven by a complex network of molecular interactions in which miR-155 and miR-21 play a central role by negatively regulating critical genes such as SOCS1, PTEN, and PIAS3.

We demonstrated that SLE patients have significantly higher levels of IL-21 in plasma and cell culture supernatant than HC. This supports the idea that IL-21 plays a key role as a soluble mediator in the inflammatory microenvironment of SLE, reflecting a persistent activation of immune cells producing this cytokine. Elevated IL-21 in plasma reflects a chronic proinflammatory state. At the same time, its increased detection in supernatants suggests that immune cells from SLE patients, even under *ex vivo* conditions, are intrinsically predisposed to produce increased amounts of this cytokine. These results are consistent with sustained activation of the STAT3 pathway in SLE and reinforce the idea that IL-21 may act as a central regulator that perpetuates immune dysfunction, both systemically and locally. One interesting aspect of our results is that IL-21 levels did not significantly correlate with clinical disease activity, Mex-SLEDAI and SLEDAI-2K, however it is important highlight that 90% of SLE patients included were inactive/mildly active. This suggests that the entire miR/STAT3/IL-21 axis is significantly dysregulated even in patients with clinically stable or inactive disease. It is plausible that IL-21 plays a more relevant role during the onset of SLE and in periods of high activity, influencing the appearance of clinical symptoms and exacerbations. However, its expression appears to have a less significant relationship with clinical manifestations in cases of long-term established SLE (78, 89). In line with these results, our team has previously demonstrated elevated IL-21 levels in SLE patients compared to HC (40, 78, 89). Previous studies have also reported elevated levels of IL-21 in plasma, serum, and cell culture supernatants in SLE patients, some of which have correlated this elevation with disease activity (63, 90, 91).

Integrating these results, our findings reveal a significant dysregulation of IL-21 expression, attributable in part to altered regulators of the STAT3 signaling pathway. This pathway, a major player in the pathogenesis of SLE, appears to be compromised by the downregulation of the regulatory molecules SOCS1, PTEN, and PIAS3, which facilitates persistent T and B cell activation. This inflammatory environment perpetuates the production of cytokines such as IL-21 and IL-17, which are relevant in the immune dysregulation characteristic of SLE. Additionally, overexpression of miRNAs, such as miR-155 and miR-21, contributes to aberrant suppression of their target genes, including STAT3 regulators, reinforcing an abnormal activation cycle in immune cells.

These findings emphasized the complexity of SLE pathogenesis and the inherent difficulties in identifying effective therapeutic targets. However, our data highlight IL-21 as a distinctive autoimmune cytokine, suggesting its potential as a biomarker and therapeutic target. It is possible that IL-21 regulation could restore homeostasis in key populations such as Tfh, Th17, and B lymphocytes, which play crucial roles in disease progression. Identifying mechanisms linking IL-21 expression and miRNAs to the STAT3 pathway opens new opportunities for developing targeted therapeutic interventions. These strategies could mitigate chronic inflammation and address the underlying immune dysfunction in SLE patients, representing a promising step toward more targeted and effective treatments.

Limitations of this study include the lack of antibody measurement, which precludes direct correlations with specific serological profiles in SLE patients. Moreover, the patients recruited for our cohort were undergoing immunomodulatory treatment, including antimalarial drugs, steroids, and azathioprine. Antimalarial drugs and steroids can alter p-STAT3 levels and modulate the expression of miR-155 and miR-21 in varying proportions, depending on the specific immune cell type (B-cells, T-cells, dendritic cells, murine models, and cancer cells) and the type of therapy (mono or combined) (92–98). Azathioprine is an antimetabolite that interferes with GTPase signaling in lymphocytes. This drug may affect signaling pathways involving p-STAT3, SOCS1, PTEN, or PIAS3; however, information on this is limited and the possible contribution should be considered but cannot be quantified with absolute certainty (99). Overall, given the prevalence of treatment in our cohort, we must interpret the differences in miR-155, miR-21, and STAT3 regulators observed here with caution, as part of the variation could be due to pharmacological effects.

Therefore, we have included this issue as a major limitation of our study and propose that future studies with naïve patients or larger sample sizes should perform stratified treatment groups to better distinguish the effects of disease versus those induced by therapy. Also, the study population is heterogeneous, with variable clinical and immunological characteristics compared to previous studies. As mRNA expression could be affected by age (100), further studies should include representative groups matched by co-founders associated with age-related inflammatory process. Consequently, the present study is limited to discuss this important phenomenon. Almost all patients were under standard of care and pharmacological treatment at the time of sampling; therefore, it was not possible to evaluate treatment influence on the interest molecules. Furthermore, although the expression of miR-155 and miR-21 was analyzed about their target genes (SOCS1, PTEN, and PIAS3), direct effect was not tested using miRNA mimics or inhibitors, nor were additional studies using RNA interference (RNAi) to silence these target genes. However, unlike other studies, this work analyzed the three genes together with their associated miRNAs at both the mRNA and protein levels. Still, evaluating them in specific cell subpopulations, such as CD4+ T cells (Tfh, Tph, and Th17) or B cells, could provide further insight into their role in disease pathogenesis. Finally, the subcellular localization of p-STAT3 was not assessed; therefore, future studies could address this aspect through nuclear and cytoplasmic fractionation followed by Western blot analysis.

## 5 Conclusion

Our findings highlight the critical role of IL-21 in the pathogenesis of SLE, showing elevated levels in plasma and after cell stimulation. This increase drives aberrant activation and proliferation of T and B cells, promoting autoantibody production. Furthermore, the overexpression of miR-155 and miR-21, combined with decreased SOCS1, PTEN, and PIAS3 levels, enhances STAT3 signaling and perpetuates chronic inflammation. These findings suggest potential therapeutic targets to address immune dysregulation in SLE.

## Data Availability

The raw data supporting the conclusions of this article will be made available by the authors, without undue reservation.

## Glossary

ACR: American College of Rheumatology
AFCs: Antibody-forming cells
Anti-dsDNA: Anti-double-stranded DNA
CD3: Cluster of differentiation 3
CD4: Cluster of differentiation 4
cTfh: Circulating T follicular helper
CXCR5: CXC chemokine receptor 5
DN: Double negative
ELISA: Enzyme-Linked ImmunoSorbent Assay
EULAR: The European Alliance of Associations for Rheumatology
FCS: Fetal Calf Serum
GAPDH: Glyceraldehyde-3-phosphate dehydrogenase
GC: Germinal centers
HCs: Healthy controls
HRP: Horseradish peroxidase
I: ionomycin
IFN: Interferon
IFN-γ: Type I interferon gamma
IQR: Interquartile range
IL-6: Interleukin 6
IL-10: Interleukin 10
IL-17A: Interleukin 17A
IL-21: Interleukin 21
IL-23: Interleukin 23
IL-21R: IL-21 receptor
IL-23R: IL-23 receptor
JAK2: Janus kinase 2
Mex-SLEDAI: Mexican version of the Systemic Lupus Erythematosus Disease Activity Index
miRNAs: MicroRNAs
miR-21: microRNA-21
miR-155: microRNA-155
miR-320a: microRNA-320a
NKT: Natural killer T
nm: nanometer
PAX5: Paired box gene 5
PBMCs: Peripheral blood mononuclear cells
pDCs: Plasmacytoid dendritic cell
PIAS3: Protein inhibitor of activated STAT3
PMA: Phorbol myristate acetate
p-STAT3: Phosphorylated STAT3
PTEN: Phosphatase and tensin homolog
PVDF: Transferred to a polyvinylidene difluoride
RT-qPCR: Reverse Transcription-Quantitative Polymerase Chain Reaction
SDS-PAGE: Sodium dodecyl sulfate polyacrylamide gel electrophoresis
SLE: Systemic lupus erythematosus
SLEDAI: Systemic Lupus Erythematosus Disease Activity Index
SLEDAI-2K: Systemic Lupus Erythematosus Disease Activity Index 2000
SLICC: Systemic Lupus International Collaborating Clinics
SOCS1: Cytokine signaling 1
STAT3: Signal transducer and activator of transcription 3
S727: Serine 727
Tfh: T follicular helper
TNF- α: Tumor necrosis factor-alpha
Tph: T peripheral helper
Th1: Type 1 helper T
Th17: T helper 17
Treg: Regulatory T
Y705: Tyrosine 705.

## References

[1] Tsokos GC . Systemic lupus erythematosus, Mechanisms of disease review article. N Engl J Med. (2011) 365:2110–21. doi: 10.1056/NEJMra1100359, PMID: 22129255

[2] Kiriakidou M Ching CL . Systemic lupus erythematosus. Ann Intern Med. (2020) 172:ITC82–96. doi: 10.7326/AITC202006020, PMID: 32479157

[3] Tsokos GC . Autoimmunity and organ damage in systemic lupus erythematosus. Nat Immunol. (2020) 21:605–14. doi: 10.1038/s41590-020-0677-6, PMID: 32367037 PMC8135909

[4] Deng XM Yan SX Wei W . IL-21 acts as a promising therapeutic target in systemic lupus erythematosus by regulating plasma cell differentiation. Cell Mol Immunol Chin Soc Immunology;. (2015) 12:31–9. doi: 10.1038/cmi.2014.58, PMID: 25088225 PMC4654374

[5] Seyedsadr M Bang MF McCarthy EC Zhang S Chen HC Mohebbi M . A pathologically expanded, clonal lineage of IL-21-producing CD4+ T cells drives inflammatory neuropathy. J Clin Invest. (2024) 134. doi: 10.1172/JCI178602, PMID: 39087473 PMC11290969

[6] Koh CH Kim BS Kang CY Chung Y Seo H . IL-17 and IL-21: their immunobiology and therapeutic potentials. Immune Netw. (2024) 24. doi: 10.4110/in.2024.24.e2, PMID: 38455465 PMC10917578

[7] Spolski R Leonard WJ . Interleukin-21: A double-edged sword with therapeutic potential. Nat Rev Drug Discov. (2014) 13:379–95. doi: 10.1038/nrd4296, PMID: 24751819

[8] Bartel DP . MicroRNAs: genomics, biogenesis, mechanism, and function. Cell. (2004) 116:281–97. doi: 10.1016/S0092-8674(04)00045-5, PMID: 14744438

[9] Khoshmirsafa M Kianmehr N Falak R Mowla SJ Seif F Mirzaei B . Elevated expression of miR-21 and miR-155 in peripheral blood mononuclear cells as potential biomarkers for lupus nephritis. Int J Rheum Dis. (2019) 22:458–67. doi: 10.1111/1756-185X.13410, PMID: 30398001

[10] Domingo S Solé C Cortés-Hernández J Moliné T Ferrer B . MicroRNAs in several cutaneous autoimmune diseases: psoriasis, cutaneous lupus erythematosus and atopic dermatitis. Cells. (2020) 9:2656. doi: 10.3390/cells9122656, PMID: 33321931 PMC7763020

[11] Ott N Faletti L Heeg M Andreani V Grimbacher B . JAKs and STATs from a clinical perspective: loss-of-function mutations, gain-of-function mutations, and their multidimensional consequences. J Clin Immunol. (2023) 43:1326–59. doi: 10.1007/s10875-023-01483-x, PMID: 37140667 PMC10354173

[12] Wang H Wang J Xia Y . Defective suppressor of cytokine signaling 1 signaling contributes to the pathogenesis of systemic lupus erythematosus. Front Immunol. (2017) 8:300780. doi: 10.3389/fimmu.2017.01292, PMID: 29085365 PMC5650678

[13] Shrestha S Yang K Guy C Vogel P Neale G Chi H . Treg cells require the phosphatase PTEN to restrain TH1 and TFH cell responses. Nat Immunol. (2015) 16:178–87. doi: 10.1038/ni.3076, PMID: 25559258 PMC4297581

[14] Lee SH Moon SJ Park MJ Kim EK Moon YM Cho ML . PIAS3 suppresses acute graft-versus-host disease by modulating effector T and B cell subsets through inhibition of STAT3 activation. Immunol Lett. (2014) 160:79–88. doi: 10.1016/j.imlet.2014.03.014, PMID: 24718277

[15] Li W Liu S Chen Y Weng R Zhang K He X . Circulating exosomal micrornas as biomarkers of systemic lupus erythematosus. Clinics. (2020) 75:1–6. doi: 10.6061/clinics/2020/e1528, PMID: 32876110 PMC7442402

[16] Ibrahim MRK Waly NG Moness H Ahmed SS Ibrahem R . Serum miRNA-21, miRNA-146a and plasma cell free DNA as novel biomarkers for assessing systemic lupus erythematosus activity. Mol Biol Rep. (2023) 50:10025–36. doi: 10.1007/s11033-023-08845-z, PMID: 37904010 PMC10676317

[17] Wu XN Ye YX Niu JW Li Y Li X You X . Defective PTEN regulation contributes to B cell hyperresponsiveness in systemic lupus erythematosus. Sci Transl Med. (2014) 6. doi: 10.1126/scitranslmed.3009131, PMID: 25101889

[18] Yu J Mei J Zuo D Zhang M Yu S Li F . Inflammatory factor-mediated miR-155/SOCS1 signaling axis leads to Treg impairment in systemic lupus erythematosus. Int Immunopharmacol. (2024) 141:113013. doi: 10.1016/j.intimp.2024.113013, PMID: 39213866

[19] Rasmussen TK Andersen T Bak RO Yiu G Sørensen CM Stengaard-Pedersen K . Overexpression of microRNA-155 increases IL-21 mediated STAT3 signaling and IL-21 production in systemic lupus erythematosus. Arthritis Res Ther. (2015) 17:154. doi: 10.1186/s13075-015-0660-z, PMID: 26055806 PMC4504038

[20] Aringer M Costenbader K Daikh D Brinks R Mosca M Ramsey-Goldman R . 2019 European league against rheumatism/american college of rheumatology classification criteria for systemic lupus erythematosus. Arthritis Rheumatol. (2019) 71:1400–12. doi: 10.1002/art.40930, PMID: 31385462 PMC6827566

[21] Guzman J Cardiel MH Arce-Salinas A Sanchez-Guerrero J Alarcon-Segovia D . Measurement of disease activity in systemic lupus erythematosus. Prospective validation of 3 clinical indices. J Rheumatol. (1992) 19:1551–8., PMID: 1464867

[22] Arora S Isenberg DA Castrejon I . Measures of adult systemic lupus erythematosus: disease activity and damage. Arthritis Care Res (Hoboken). (2020) 72:27–46. doi: 10.1002/acr.24221, PMID: 33091256

[23] Gladman D Ginzler E Goldsmith C Fortin P Liang M Urowitz M . The development and initial validation of the Systemic Lupus International Collaborating Clinics/American College of Rheumatology damage index for systemic lupus erythematosus. Arthritis Rheum. (1996) 39:363–9. doi: 10.1002/art.1780390303, PMID: 8607884

[24] World Medical Asociation (AMM) . World Medical Association declaration of Helsinki: Ethical principles for medical research involving human subjects. JAMA. (2013) 310:2191–4. doi: 10.1001/jama.2013.281053, PMID: 24141714

[25] El-Shaer OS Sabry JH Mahgoub MY Hamed NA Nour El Din DM Ameen SG . MiR-155 expression is a potential biomarker of systemic lupus erythematosus diagnosis and disease activity prediction. Meta Gene. (2020) 26:100770. doi: 10.1016/j.mgene.2020.100770

[26] Gao X Song Y Wu J Lu S Min X Liu L . Iron-dependent epigenetic modulation promotes pathogenic T cell differentiation in lupus. J Clin Invest. (2022) 132. doi: 10.1172/JCI159472, PMID: 35499082 PMC9057600

[27] Stypinska B Wajda A Walczuk E Olesinska M Lewandowska A Walczyk M . The serum cell-free microRNA expression profile in MCTD, SLE, SSc, and RA patients. J Clin Med. (2020) 9:161. doi: 10.3390/jcm9010161, PMID: 31936082 PMC7020053

[28] Fedeli M Kuka M Finardi A Albano F Viganò V Iannacone M . miR-21 sustains CD28 signalling and low-affinity T-cell responses at the expense of self-tolerance. Clin Transl Immunol. (2021) 10:e1321. doi: 10.1002/cti2.1321, PMID: 34584693 PMC8454917

[29] Kim C Hu B Jadhav RR Jin J Zhang H Cavanagh MM . Activation of miR-21-Regulated Pathways in Immune Aging Selects against Signatures Characteristic of Memory T Cells. Cell Rep. (2018) 25:2148–2162.e5. doi: 10.1016/j.celrep.2018.10.074, PMID: 30463012 PMC6371971

[30] Kunze-Schumacher H Winter SJ Imelmann E Krueger A . MiRNA miR-21 is largely dispensable for intrathymic T-cell development. Front Immunol. (2018) 9:420522. doi: 10.3389/fimmu.2018.02497, PMID: 30455689 PMC6230590

[31] Zhu F Li H Liu Y Tan C Liu X Fan H . miR-155 antagomir protect against DSS-induced colitis in mice through regulating Th17/Treg cell balance by Jarid2/Wnt/β-catenin. Biomedicine Pharmacotherapy. (2020) 126:109909. doi: 10.1016/j.biopha.2020.109909, PMID: 32135463

[32] Paoletti A Rohmer J Ly B Pascaud J Rivière E Seror R . Monocyte/macrophage abnormalities specific to rheumatoid arthritis are linked to miR-155 and are differentially modulated by different TNF inhibitors. J Immunol. (2019) 203:1766–75. doi: 10.4049/jimmunol.1900386, PMID: 31484730 PMC6755128

[33] Liu Y Dong Y Zhu X Fan H Xu M Chen Q . MiR-155 inhibition ameliorates 2, 4, 6-Trinitrobenzenesulfonic acid (TNBS)-induced experimental colitis in rat via influencing the differentiation of Th17 cells by Jarid2. Int Immunopharmacol. (2018) 64:401–10. doi: 10.1016/j.intimp.2018.09.007, PMID: 30253332

[34] Goncalves-Alves E Saferding V Schliehe C Benson R Kurowska-Stolarska M Brunner JS . MicroRNA-155 controls T helper cell activation during viral infection. Front Immunol. (2019) 10:1367. doi: 10.3389/fimmu.2019.01367, PMID: 31275315 PMC6593301

[35] Hu R Kagele DA Huffaker TB Runtsch MC Alexander M Liu J . MiR-155 promotes T follicular helper cell accumulation during chronic, low-grade inflammation. Immunity. (2014) 41:605–19. doi: 10.1016/j.immuni.2014.09.015, PMID: 25367574 PMC4657560

[36] Lu D Nakagawa R Lazzaro S Staudacher P Abreu-Goodger C Henley T . The miR-155-PU.1 axis acts on Pax5 to enable efficient terminal B cell differentiation. J Exp Med. (2014) 211:2183–98. doi: 10.1084/jem.20140338, PMID: 25288398 PMC4203942

[37] Arbore G Henley T Biggins L Andrews S Vigorito E Turner M . MicroRNA-155 is essential for the optimal proliferation and survival of plasmablast B cells. Life Sci Alliance. (2019) 2. doi: 10.26508/lsa.201800244, PMID: 31097471 PMC6524163

[38] Chauhan SK Singh VV Rai R Rai M Rai G . Differential microRNA profile and post-transcriptional regulation exist in systemic lupus erythematosus patients with distinct autoantibody specificities. J Clin Immunol. (2014) 34:491–503. doi: 10.1007/s10875-014-0008-5, PMID: 24659206

[39] Chen JQ Papp G Póliska S Szabó K Tarr T Bálint BL . MicroRNA expression profiles identify disease-specific alterations in systemic lupus erythematosus and primary Sjögren’s syndrome. PloS One. (2017) 12:e0174585. doi: 10.1371/journal.pone.0174585, PMID: 28339495 PMC5365120

[40] Álvarez Gómez JA Salazar-Camarena DC Román-Fernández IV Ortiz-Lazareno PC Cruz A Muñoz-Valle JF . BAFF system expression in double negative 2, activated naïve and activated memory B cells in systemic lupus erythematosus. Front Immunol. (2023) 14:1235937. doi: 10.3389/fimmu.2023.1235937, PMID: 37675114 PMC10478082

[41] Zhao Q Huang L Qin G Qiao Y Ren F Shen C . Cancer-associated fibroblasts induce monocytic myeloid-derived suppressor cell generation via IL-6/exosomal miR-21-activated STAT3 signaling to promote cisplatin resistance in esophageal squamous cell carcinoma. Cancer Lett. (2021) 518:35–48. doi: 10.1016/j.canlet.2021.06.009, PMID: 34139285

[42] Salvi V Gianello V Busatto S Bergese P Andreoli L D’Oro U . Exosome-delivered microRNAs promote IFN-α secretion by human plasmacytoid DCs via TLR7. JCI Insight. (2018) 3. doi: 10.1172/jci.insight.98204, PMID: 29769437 PMC6012509

[43] Schell SL Bricker KN Fike AJ Chodisetti SB Domeier PP Choi NM . Context-dependent miR-21 regulation of TLR7-mediated autoimmune and foreign antigen–driven antibody-forming cell and germinal center responses. J Immunol. (2021) 206:2803–18. doi: 10.4049/jimmunol.2001039, PMID: 34039637 PMC8617059

[44] Sukka-Ganesh B Larkin J . Therapeutic potential for targeting the suppressor of cytokine signalling-1 pathway for the treatment of SLE. Scand J Immunol. (2016) 84:299–309. doi: 10.1111/sji.12475, PMID: 27781323 PMC5131794

[45] Qiu LJ Xu K Liang Y Cen H Zhang M Wen PF . Decreased SOCS1 mRNA expression levels in peripheral blood mononuclear cells from patients with systemic lupus erythematosus in a Chinese population. Clin Exp Med. (2015) 15:261–7. doi: 10.1007/s10238-014-0309-2, PMID: 25330931

[46] Guo H Li R Wang M Hou Y Liu S Peng T . Multiomics analysis identifies SOCS1 as restraining T cell activation and preventing graft-versus-host disease. Advanced Sci. (2022) 9:2200978. doi: 10.1002/advs.202200978, PMID: 35585676 PMC9313503

[47] Kamran MZ Patil P Gude RP . Role of STAT3 in cancer metastasis and translational advances. BioMed Res Int. (2013) 2013. doi: 10.1155/2013/421821, PMID: 24199193 PMC3807846

[48] Yoshida N He F Kyttaris VC . T cell–specific STAT3 deficiency abrogates lupus nephritis. Lupus. (2019) 28:1468–72. doi: 10.1177/0961203319877242, PMID: 31551033 PMC6791775

[49] Takahashi R Nishimoto S Muto G Sekiya T Tamiya T Kimura A . SOCS1 is essential for regulatory T cell functions by preventing loss of Foxp3 expression as well as IFN-γ and IL-17A production. J Exp Med. (2011) 208:2055–67. doi: 10.1084/jem.20110428, PMID: 21893603 PMC3182063

[50] Sun S Steinberg BM . PTEN is a negative regulator of STAT3 activation in human papillomavirus-infected cells. J Gen Virol. (2002) 83:1651–8. doi: 10.1099/0022-1317-83-7-1651, PMID: 12075083

[51] Lee SH Park JS Byun JK Jhun J Jung K Seo HB . PTEN ameliorates autoimmune arthritis through down-regulating STAT3 activation with reciprocal balance of Th17 and Tregs. Sci Rep. (2016) 6. doi: 10.1038/srep34617, PMID: 27708408 PMC5052580

[52] Cui D Zhu D Ren H Lin J Lai W Huang Q . MicroRNA-198 contributes to lupus nephritis progression by inhibition of phosphatase and tensin homology deleted on chromosome ten expression. Mol Med Rep. (2017) 16:7813–20. doi: 10.3892/mmr.2017.7527, PMID: 28944868

[53] Wu S Wang J Li F . Dysregulation of PTEN caused by the underexpression of microRNA-130b is associated with the severity of lupus nephritis. Mol Med Rep. (2018) 17:7966–72. doi: 10.3892/mmr.2018.8839, PMID: 29620214

[54] Kim HS Jang SW Lee W Kim K Sohn H Hwang SS . PTEN drives Th17 cell differentiation by preventing IL-2 production. J Exp Med. (2017) 214:3381–98. doi: 10.1084/jem.20170523, PMID: 29018045 PMC5679178

[55] Kluge A Dabir S Vlassenbroeck I Eisenberg R Dowlati A . Protein inhibitor of activated STAT3 expression in lung cancer. Mol Oncol. (2011) 5:256–64. doi: 10.1016/j.molonc.2011.03.004, PMID: 21497567 PMC3104085

[56] Tang X Yin K Zhu H Tian J Shen D Yi L . Correlation between the expression of microRNA-301a-3p and the proportion of th17 cells in patients with rheumatoid arthritis. Inflammation. (2016) 39:759–67. doi: 10.1007/s10753-016-0304-8, PMID: 26782362

[57] Seko Y Cole S Kasprzak W Shapiro BA Ragheb JA . The role of cytokine mRNA stability in the pathogenesis of autoimmune disease. Autoimmun Rev. (2006) 5:299–305. doi: 10.1016/j.autrev.2005.10.013, PMID: 16782553

[58] Schwanhüusser B Busse D Li N Dittmar G Schuchhardt J Wolf J . Global quantification of mammalian gene expression control. Nature. (2011) 473:337–42. doi: 10.1038/nature10098, PMID: 21593866

[59] Cichanover A . Intracellular protein degradation: From a vague idea thru the lysosome and the ubiquitin-proteasome system and onto human diseases and drug targeting. Cell Death Differentiation. (2005) 12:1178–90. doi: 10.1038/sj.cdd.4401692, PMID: 16094394

[60] Caprioli F Sarra M Caruso R Stolfi C Fina D Sica G . Autocrine regulation of IL-21 production in human T lymphocytes. J Immunol. (2008) 180:1800–7. doi: 10.4049/jimmunol.180.3.1800, PMID: 18209077

[61] Dolff S Abdulahad WH Westra J Doornbos-van der Meer B Limburg PC Kallenberg CGM . Increase in IL-21 producing T-cells in patients with systemic lupus erythematosus. Arthritis Res Ther. (2011) 13:R157. doi: 10.1186/ar3474, PMID: 21959034 PMC3308088

[62] Lee J Shin EK Lee SY Her YM Park MK Kwok SK . Oestrogen up-regulates interleukin-21 production by CD4+ T lymphocytes in patients with systemic lupus erythematosus. Immunology. (2014) 142:573–80. doi: 10.1111/imm.12263, PMID: 24495300 PMC4107667

[63] Yang H Liang N Wang M Fei Y Sun J Li Z . Long noncoding RNA MALAT-1 is a novel inflammatory regulator in human systemic lupus erythematosus. Oncotarget. (2017) 8:77400–6. doi: 10.18632/oncotarget.20490, PMID: 29100395 PMC5652787

[64] Nakou M Papadimitraki ED Fanouriakis A Bertsias GK Choulaki C Goulidaki N . Interleukin-21 is increased in active systemic lupus erythematosus patients and contributes to the generation of plasma B cells. Clin Exp Rheumatol. (2013) 31:0172–9., PMID: 23137515

[65] Shi Y Chen Z Zhao Z Yu Y Fan H Xu X . IL-21 induces an imbalance of Th17/treg cells in moderate-to-severe plaque psoriasis patients. Front Immunol. (2019) 10:1865. doi: 10.3389/fimmu.2019.01865, PMID: 31440249 PMC6693306

[66] Nurieva R Yang XO Martinez G Zhang Y Panopoulos AD Ma L . Essential autocrine regulation by IL-21 in the generation of inflammatory T cells. Nature. (2007) 448:480–3. doi: 10.1038/nature05969, PMID: 17581589

[67] Zhang J Ren M Zeng H Guo Y Zhuang Z Feng Z . Elevated follicular helper T Cells and expression of IL-21 in thyroid tissues are involved in the pathogenesis of Graves’ disease. Immunol Res. (2015) 62:163–74. doi: 10.1007/s12026-015-8647-z, PMID: 25894310

[68] Wong CK Wong PTY Tam LS Li EK Chen DP Lam CWK . Elevated production of B Cell Chemokine CXCL13 is correlated with systemic lupus erythematosus disease activity. J Clin Immunol. (2010) 30:45–52. doi: 10.1007/s10875-009-9325-5, PMID: 19774453

[69] Wang S Wang J Kumar V Karnell JL Naiman B Gross PS . IL-21 drives expansion and plasma cell differentiation of autoreactive CD11chiT-bet+ B cells in SLE. Nat Commun. (2018) 9:1–14. doi: 10.1038/s41467-018-03750-7, PMID: 29717110 PMC5931508

[70] Kang KY Kim HO Kwok SK Ju JH Park KS Sun D . Impact of interleukin-21 in the pathogenesis of primary Sjögren’s syndrome: increased serum levels of interleukin-21 and its expression in the labial salivary glands. Arthritis Res Ther. (2011) 13:R179. doi: 10.1186/ar3504, PMID: 22030011 PMC3308114

[71] Quast I Dvorscek AR Pattaroni C Steiner TM McKenzie CI Pitt C . Interleukin-21, acting beyond the immunological synapse, independently controls T follicular helper and germinal center B cells. Immunity. (2022) 55:1414–1430.e5. doi: 10.1016/j.immuni.2022.06.020, PMID: 35896116

[72] Herber D Brown TP Liang S Young DA Collins M Dunussi-Joannopoulos K . IL-21 has a pathogenic role in a lupus-prone mouse model and its blockade with IL-21R.Fc reduces disease progression. J Immunol. (2007) 178:3822–30. doi: 10.4049/jimmunol.178.6.3822, PMID: 17339481

[73] Nguyen V Luzina I Rus H Tegla C Chen C Rus V . IL-21 promotes lupus-like disease in chronic graft-versus-host disease through both CD4 T cell- and B cell-intrinsic mechanisms. J Immunol. (2012) 189:1081–93. doi: 10.4049/jimmunol.1200318, PMID: 22723520 PMC3392550

[74] Korn T Bettelli E Oukka M Kuchroo VK . IL-17 and th17 cells. Annu Rev Immunol. (2009) 27:485–517. doi: 10.1146/annurev.immunol.021908.132710, PMID: 19132915

[75] Park H Li Z Yang XO Chang SH Nurieva R Wang YH . A distinct lineage of CD4 T cells regulates tissue inflammation by producing interleukin 17. Nat Immunol. (2005) 6:1133–41. doi: 10.1038/ni1261, PMID: 16200068 PMC1618871

[76] Korn T Bettelli E Gao W Awasthi A Jäger A Strom TB . IL-21 initiates an alternative pathway to induce proinflammatory T H17 cells. Nature. (2007) 448:484–7. doi: 10.1038/nature05970, PMID: 17581588 PMC3805028

[77] Qin H Wang L Feng T Elson CO Niyongere SA Lee SJ . TGF-β Promotes th17 cell development through inhibition of SOCS3. J Immunol. (2009) 183:97–105. doi: 10.4049/jimmunol.0801986, PMID: 19535626 PMC2851540

[78] Sagrero-Fabela N Ortíz-Lazareno PC Salazar-Camarena DC Cruz A Cerpa-Cruz S Muñoz-Valle JF . BAFFR expression in circulating T follicular helper (CD4+CXCR5+PD-1+) and T peripheral helper (CD4+CXCR5–PD-1+) cells in systemic lupus erythematosus. Lupus. (2023) 32:1093–104. doi: 10.1177/09612033231189804, PMID: 37460408

[79] Ding J Su S You T Xia T Lin X Chen Z . Serum interleukin-6 level is correlated with the disease activity of systemic lupus erythematosus: a meta-analysis. Clinics. (2020) 75:e1801. doi: 10.6061/clinics/2020/e1801, PMID: 33084768 PMC7536892

[80] Geginat J Vasco M Gerosa M Tas SW Pagani M Grassi F . IL-10 producing regulatory and helper T-cells in systemic lupus erythematosus. Semin Immunol. (2019) 44:101330. doi: 10.1016/j.smim.2019.101330, PMID: 31735515

[81] Avery DT Deenick EK Ma CS Suryani S Simpson N Chew GY . B cell-intrinsic signaling through IL-21 receptor and STAT3 is required for establishing long-lived antibody responses in humans. J Exp Med. (2010) 207:155–71. doi: 10.1084/jem.20091706, PMID: 20048285 PMC2812540

[82] Dai H He F Tsokos GC Kyttaris VC . IL-23 limits the production of IL-2 and promotes autoimmunity in lupus. J Immunol. (2017) 199:903–10. doi: 10.4049/jimmunol.1700418, PMID: 28646040 PMC5526729

[83] Dean EC Ditoro DF Pham D Gao M Zindl CL Frey B . IL-2–induced stat3 signaling is critical for effector treg cell programming. bioRxiv. (2023). doi: 10.1101/2023.09.26.559434v1, PMID: 37808649

[84] Harada T Kyttaris V Li Y Juang YT Wang Y Tsokos GC . Increased expression of STAT3 in SLE T cells contributes to enhanced chemokine-mediated cell migration. Autoimmunity. (2007) 40:1–8. doi: 10.1080/08916930601095148, PMID: 17364491

[85] Hedrich CM Rauen T Apostolidis SA Grammatikos AP Rodriguez NR Ioannidis C . Stat3 promotes IL-10 expression in lupus T cells through trans-activation and chromatin remodeling. Proc Natl Acad Sci U.S.A. (2014) 111:13457–62. doi: 10.1073/pnas.1408023111, PMID: 25187566 PMC4169908

[86] Wan CK Andraski AB Spolski R Li P Kazemian M Oh J . Opposing roles of STAT1 and STAT3 in IL-21 function in CD4+ T cells. Proc Natl Acad Sci U.S.A. (2015) 112:9394–9. doi: 10.1073/pnas.1511711112, PMID: 26170288 PMC4522759

[87] Zhang Y Jing Z Cao X Wei Q He W Zhang N . SOCS1, the feedback regulator of STAT1/3, inhibits the osteogenic differentiation of rat bone marrow mesenchymal stem cells. Gene. (2022) 821:146190. doi: 10.1016/j.gene.2022.146190, PMID: 35124149

[88] Hale MB Krutzik PO Samra SS Crane JM Nolan GP . Stage dependent aberrant regulation of cytokine-STAT signaling in murine systemic lupus erythematosus. PloS One. (2009) 4. doi: 10.1371/journal.pone.0006756, PMID: 19707593 PMC2727051

[89] Espinoza-García N Salazar-Camarena DC Marín-Rosales M Reyes-Mata MP Ramírez-Dueñas MG Muñoz-Valle JF . High interleukin 21 levels in patients with systemic lupus erythematosus: association with clinical variables and rs2221903 polymorphism. J Clin Med. (2024) 13. doi: 10.3390/jcm13154512, PMID: 39124778 PMC11313274

[90] Singh RP Hahn BH Bischoff DS . Interferon genes are influenced by 17β-estradiol in SLE. Front Immunol. (2021) 12:725325. doi: 10.3389/fimmu.2021.725325, PMID: 34733276 PMC8558410

[91] Shater H Fawzy M Farid A El-Amir A Fouad S Madbouly N . The potential use of serum interleukin-21 as biomarker for lupus nephritis activity compared to cytokines of the tumor necrosis factor (TNF) family. Lupus. (2022) 31:55–64. doi: 10.1177/09612033211063794, PMID: 34978958

[92] Mazloumfard F Mirian M Eftekhari SM Aliomrani M . Hydroxychloroquine effects on miR-155-3p and miR-219 expression changes in animal model of multiple sclerosis. Metab Brain Dis. (2020) 35:1299–307. doi: 10.1007/s11011-020-00609-z, PMID: 32860610

[93] Chafin CB Regna NL Hammond SE Reilly CM . Cellular and urinary microRNA alterations in NZB/W mice with hydroxychloroquine or prednisone treatment. Int Immunopharmacol. (2013) 17:894–906. doi: 10.1016/j.intimp.2013.09.013, PMID: 24121037 PMC3868221

[94] Chen C Zhang H Yu Y Huang Q Wang W Niu J . Chloroquine suppresses proliferation and invasion and induces apoptosis of osteosarcoma cells associated with inhibition of phosphorylation of STAT3. Aging. (2021) 13:17901–13. doi: 10.18632/aging.203196, PMID: 34170850 PMC8312460

[95] El-Maadawy WH Hafiz E Tadros SA Fahim SA Ebrahim HM Fouad MA . Hydroxychloroquine modulates the progression of experimentally induced benign prostatic hyperplasia in rats via targeting EGFR/ERK/STAT3 and AR/FOXO1/TRAIL pathways: computational and *in vivo* studies. Sci Rep. (2025) 15:20118. doi: 10.1038/s41598-025-04267-y, PMID: 40541965 PMC12181434

[96] Thome R Bonfanti AP Rasouli J Mari ER Zhang GX Rostami A . Chloroquine-treated dendritic cells require STAT1 signaling for their tolerogenic activity. Eur J Immunol. (2018) 48:1228–34. doi: 10.1002/eji.201747362, PMID: 29572810 PMC6473787

[97] Choi DS Blanco E Kim YS Rodriguez AA Zhao H Huang THM . Chloroquine eliminates cancer stem cells through deregulation of Jak2 and DNMT1. Stem Cells. (2014) 32:2309–23. doi: 10.1002/stem.1746, PMID: 24809620 PMC4138251

[98] Davis TE Kis-Toth K Tsokos GC . A114: methylprednisolone-induced inhibition of miR-155 expression increases SOCS1-driven suppression of cytokine signaling. Arthritis Rheumatol. (2014) 66:S151–1. doi: 10.1002/art.38535

[99] Poppe D Tiede I Fritz G Becker C Bartsch B Wirtz S . Azathioprine suppresses ezrin-radixin-moesin-dependent T cell-APC conjugation through inhibition of vav guanosine exchange activity on rac proteins. J Immunol. (2006) 176:640. doi: 10.4049/jimmunol.176.1.640, PMID: 16365460 PMC1965586

[100] Schneider J Preyer C Steil M Biazid M Pointner A Haslberger AG . MiRNA-3Age: a microRNA-based biological age model and its modulation by lifestyle and nutrition. Front Nutr. (2025) 12:1659730. doi: 10.3389/fnut.2025.1659730, PMID: 41356832 PMC12677062

